# 3D spheroid cultures improve the metabolic gene expression profiles of HepaRG cells

**DOI:** 10.1042/BSR20150034

**Published:** 2015-06-11

**Authors:** Yu Takahashi, Yuji Hori, Tomohisa Yamamoto, Toshiki Urashima, Yasunori Ohara, Hideo Tanaka

**Affiliations:** * Japan Tobacco Inc., Central Pharmaceutical Research Institute, 1-1 Murasaki-cho, Takatsuki, Osaka 569-1125, Japan

**Keywords:** 3D culture, gene expression, hanging drop, HepG2 cells, HepaRG cells

## Abstract

3D (three-dimensional) cultures are considered to be an effective method for toxicological studies; however, little evidence has been reported whether 3D cultures have an impact on hepatocellular physiology regarding lipid or glucose metabolism. In the present study, we conducted physiological characterization of hepatoma cell lines HepG2 and HepaRG cells cultured in 3D conditions using a hanging drop method to verify the effect of culture environment on cellular responses. Apo (Apolipoprotein)B as well as albumin secretion was augmented by 3D cultures. Expression of genes related to not only drug, but also glucose and lipid metabolism were significantly enhanced in 3D cultured HepaRG spheroids. Furthermore, mRNA levels of CYP (cytochrome P450) enzymes following exposure to corresponding inducers increased under the 3D condition. These data suggest that this simple 3D culture system without any special biomaterials can improve liver-specific characteristics including lipid metabolism. Considering that the system enables high-throughput assay, it may become a powerful tool for compound screening concerning hepatocellular responses in order to identify potential drugs.

## Abstract (precis)

Apo (Apolipoprotein)B secretion, as well as albumin secretion, increased in 3D HepG2 and HepaRG spheroids. Liver metabolic gene expression was up-regulated in 3D HepaRG spheroids. These results suggest that hanging drop 3D cultures can improve hepatocellular responses as a functional liver.

## INTRODUCTION

Recently, the demand to establish physiological assays has increased, in particular for compound screening and drug development [1,2]. Cell-based assays are essential for validating the pharmacological activity of drug candidates; however, development for a large number of these candidates is often discontinued during subsequent animal testing or clinical trials, which entails significant commitments in terms of costs, time and energy. One of the reasons for these failures is poor predictability associated with monolayer 2D (two-dimensional) cultures not mimicking physiological conditions [3].

3D (three-dimensional) cultures, which may reflect the *in vivo* environment, are regarded as an effective method for cancer research and toxicological studies [4–6]. Some reports indicate different cellular responses for drug toxicities between 2D and 3D conditions [7–9]. There are numerous commercial 3D culture systems available for multiple applications using special biomaterials such as collagen [10], hyaluronic acid [11], methylcellulose [12] and poly-2-hydroxyethyl methacrylate [13]. Insphero AG supplies a simple scaffold-free system in a 96-well plate format by culturing cells in hanging droplets [9,14]. The cells are assembled by gravity force and migrate with polarity and an extensive extracellular matrix including collagen is formed within the spheroids as *in vivo* tissues. When it comes to HepG2 spheroids, there are also canaliculi-like structures observed by SEM (scanning electron microscope) [14]. One of the advantages of this technology is that it does not require any special materials or equipment. Candidate screening to identify apoptosis inducers has reportedly been performed using this system [15]. However, studies on hepatic function, such as lipid metabolism, have not been reported to date.

PHHs (primary human hepatocytes) are considered to reflect hepatocellular activity *in vivo*; however, use of these cells is limited due to low availability. Metabolic profiles of these cells also differ significantly depending on donor source and preparation procedures [16]. HepaRG cells are derived from one of the human hepatic cell lines with more characteristics similar to PHHs compared with HepG2 cells [17]. Unlike PHHs, HepaRG cells have a stable phenotype in cultured plates and lot-to-lot variation is known to be low [18,19]. Therefore, HepaRG cells are considered to be a good alternative to PHHs in drug metabolism and toxicity studies [19] and may also be useful in studies of hepatic function or pharmacodynamics in a physiological environment.

In the present study, we examined whether some of liver-specific characters, including protein secretions and metabolic gene expression profiles, are influenced when HepaRG and/or HepG2 cells are cultured in 3D conditions. We adopted the Insphero's 96-well plate with a view to applying this technique to drug discovery in the future, since this plate can uniformly provide one spheroid per well in a high-throughput manner. By using this plate, we showed that secretions of apo (apolipoprotein) B, a major component of VLDL (very low-density lipoprotein) and LDL (low-density lipoprotein), as well as albumin, a functional liver marker, substantially increased in the 3D cultured spheroids. Moreover, we found that the expression of genes involved in not only drug and glucose metabolism, but also lipid metabolism, such as fatty acid synthesis, triglyceride synthesis, bile acid metabolism and lipoprotein production, significantly increased in the 3D HepaRG spheroids. These results indicate that hanging drop 3D cultures can enhance hepatocellular characteristics, which is expected to lead the improvement of liver-specific metabolic activities. This technology eventually may become a suitable tool to perform functional and pharmacological studies for drug development.

## MATERIALS AND METHODS

### Cell culture

Human hepatoma cell lines, HepG2 and HepaRG cells, were obtained from A.T.C.C. (American type culture collection) and KAC respectively. HepG2 cells were maintained in DMEM (Dulbecco's modified Eagle's medium) supplemented with 10% FBS, 100 units/ml penicillin and 100 μg/ml streptomycin. Passages were performed every 2–3 days. Cryopreserved differentiated HepaRG cells were maintained according to procedures from the supplier. Briefly, cells were thawed and cultured in Williams' Media E with Thaw, Plate & General Purpose Medium supplement (Life Technologies) for the first 3 days and then cultured in Williams' Media E with Maintenance/Metabolism Medium supplement (Life Technologies) in subsequent days. Media was replaced every 2 days. All cultures were performed at 37°C in 95% humidity with 5% CO_2_.

### Organotypic 3D cultures

Hanging drop 3D cultures were performed using GravityPLUS systems (Insphero). Based on recommendations from the plate manufacturer to perform initial seeding of growing cells starting at from 250 cells/well and to perform seeding of non-proliferating cells at a range between 2500 and 25000 cells, 40 μl of cell suspension was seeded into each well (from 250 to 2000 cells/well for HepG2 cells, 8000 and 24000 cells/well for HepaRG cells) of the 3D 96-well plates (GravityPLUS plates). Three quarters of the media was changed every 1–3 days. Media was replaced twice repeatedly prior to the quantification of secreted proteins. Spheroids were collected in GravityTRAP plates by adding 70 μl of fresh media to each well of the GravityPLUS plates.

### Monolayer 2D cultures

Monolayer 2D cultures were performed using non-treated 96-well plates for HepG2 cells or collagen-coated 96-well plates (BD) for HepaRG cells. Similar to the procedure for 3D plates and based on recommendations from the plate manufacturer, HepG2 cells were seeded at from 250 cells/100 μl/well in 2D 96-well plates. Comparatively, according to procedures from the supplier of HepaRG cells, HepaRG cells were seeded at 72000 cells/100 μl/well. Media was replaced every 1–3 days.

### Quantification of albumin and apoB

Before 24 h elapsed since the start of the experiments, cells were refed with fresh media. For quantification of apoB secretion, media was replaced with secretion stimulating media (DMEM with 1% BSA and 0.2 mM linoleic acid-oleic acid-albumin (Sigma)). After 24 h of culture, media and cells were separately recovered. For 3D cultures, 30 μl of media was collected initially and spheroids were subsequently collected in GravityTRAP plates by adding 100 μl of fresh media to each well of the GravityPLUS plates. Concentrations of secreted albumin and apoB were relatively measured using ELISA kits (TaKaRa) and HTRF (homogeneous time-resolved fluorescence) kits (Cisbio) respectively. Assays were performed according to instructions from each manufacturer. Each quantitative value was normalized to the corresponding viable cell signal measured by CellTiter-Glo (Promega).

### Quantitative RT-PCR

Cells or spheroids were pooled [*n* = 12 (2D) or *n* = 96 (3D)] and harvested at indicated days after seeding. Total cellular RNA was extracted using an RNA preparation kit (RNeasy Mini Kit, Qiagen) and the subsequent RT (reverse transcription) was performed using High-Capacity cDNA Reverse Transcription Kits (Applied Biosystems). Fluorescent quantitative PCR was performed on an ABI PRISM 7900 system using TaqMan Gene Expression Assays (Applied Biosystems). 18s rRNA protein transcripts were used as an internal control to normalize mRNA levels of each gene.

### Statistical analysis

The results obtained in the present study are presented as means ± S.E.M. Data were evaluated using Student's *t* test for two groups.

## RESULTS

### Increase in albumin secretion in 3D cultures

The purpose of the study is to investigate whether 3D hanging drop cultures can affect the nature of hepatic cells. Relative albumin levels secreted into culture media within 24 h were measured first and results showed that the albumin levels from viable cells were higher in 3D cultured HepG2 spheroids at any cell count (Figure 1A). Results in HepaRG cells also indicate that higher albumin secretion per viable cell was detected in 3D cultures compared with 2D cultures (Figure 1B), in which the number of cells was optimized to cover around 80% of the plates. According to the manufacturer's instruction described in ‘Materials and Methods’, less than 25000 cells/40 μl/well were used in the 3D plates. Although the total cell number (24000 cells/well) is smaller than that in 2D cultures (72000 cells/well), we consider each cell density is comparable (720 cells/μL in 2D and 600 cells/μL in 3D) and normalizing to viable cell signals enables fair comparison between 2D and 3D. Additionally, 3D cultured HepaRG spheroids which consist of 8000 cells/well also showed significantly higher albumin production per viable cell than 2D cultured cells (result not shown).

**Figure 1:**
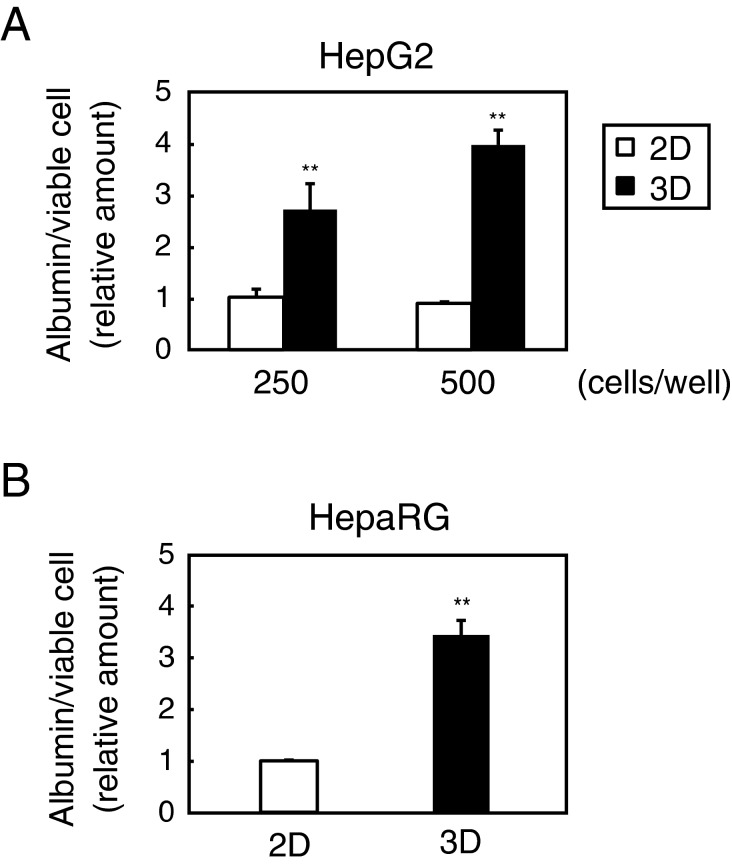
Albumin secretion increased in 3D spheroids (**A**) HepG2 cells were seeded into 96-well plates at 250 and 500 cells/well. After 5 days of culture, cells were refed with fresh media and subsequently cultured for 24 h. Media and cells were then separately collected and relative amounts of albumin and viable cell signals were respectively measured as described in the ‘Materials and Methods’ section. Error bars indicate ± S.E.M. (*n* = 4). **P<0.01, compared with 2D cultures at each cell count. (**B**) HepaRG cells were seeded into 96-well plates at 72000 cells/well (2D) or 24000 cells/well (3D) and cultured according to the procedures listed in the ‘Materials and Methods’ section. After 6 days of culture, cells were refed with fresh media and processed in the same manner as (**A**). Error bars indicate ± S.E.M. (*n* = 6). **P<0.01, compared with 2D cultures.

### Increase in apoB secretion in 3D cultures

ApoB secretion, which reflects VLDL secretion and is regarded as one of major liver functions, was next monitored. The results showed that relative amounts of apoB secretion from viable cells increased in 3D cultures at any cell count as well as albumin (Figure 2A). In HepaRG cells, the results also indicated significantly higher apoB secretion from 3D cultured spheroids both of 8000 (result not shown) and of 24000 cells/well (Figure 2B).

**Figure 2:**
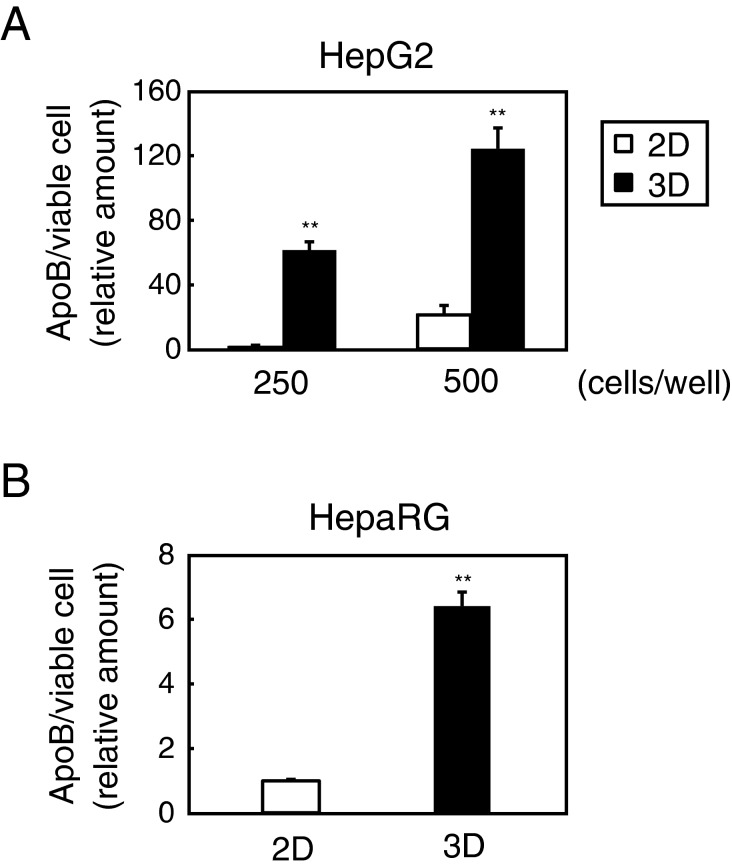
ApoB secretion increased in 3D spheroids (**A**) HepG2 cells were seeded into 96-well plates at 250 and 500 cells/well. After 6 days of culture, cells were refed with stimulating media and subsequently cultured for 24 h. Media and cells were then separately collected and relative amounts of apoB and viable cell signals were respectively measured as described in the ‘Materials and Methods’ section. Error bars indicate ± S.E.M. (*n* = 4). **P<0.01, compared with 2D cultures at each cell count. (**B**) HepaRG cells were seeded into 96-well plates at 72000 cells/well (2D) or 24000 cells/well (3D) and cultured according to the procedures listed in the ‘Materials and Methods’ section. After 6 days of culture, cells were refed with fresh media and processed in the same manner as (**A**). Error bars indicate ± S.E.M. (*n* = 6). **P<0.01, compared with 2D cultures.

### Increase in liver-specific gene expression in 3D HepaRG spheroids

Furthermore, whether liver-enriched gene expression would be affected when cells were cultured in 3D conditions was examined by quantitative RT-PCR. Unlike HepaRG cells, the gene expression of CYP (cytochrome P450) family enzymes are reported to be significantly lower in HepG2 cells than in PHHs [20]. Therefore we verified whether CYP expression is augmented in 3D cultured HepG2 spheroids. Contrary to expectations, the expression remained almost unchanged and the levels were significantly lower when compared with results obtained in 3D cultured HepaRG spheroids (Figure 3A; result not shown). Hypothesizing this as being due to HepG2 cells having limited potential as functional liver cells, it was anticipated that further optimizations of culture conditions did not aid in the enhancement of the gene expressions up to physiological levels. Hence, the focus of the present study was switched to HepaRG cells and gene expression during the time course of the culture was compared between 2D and 3D conditions.

**Figure 3:**
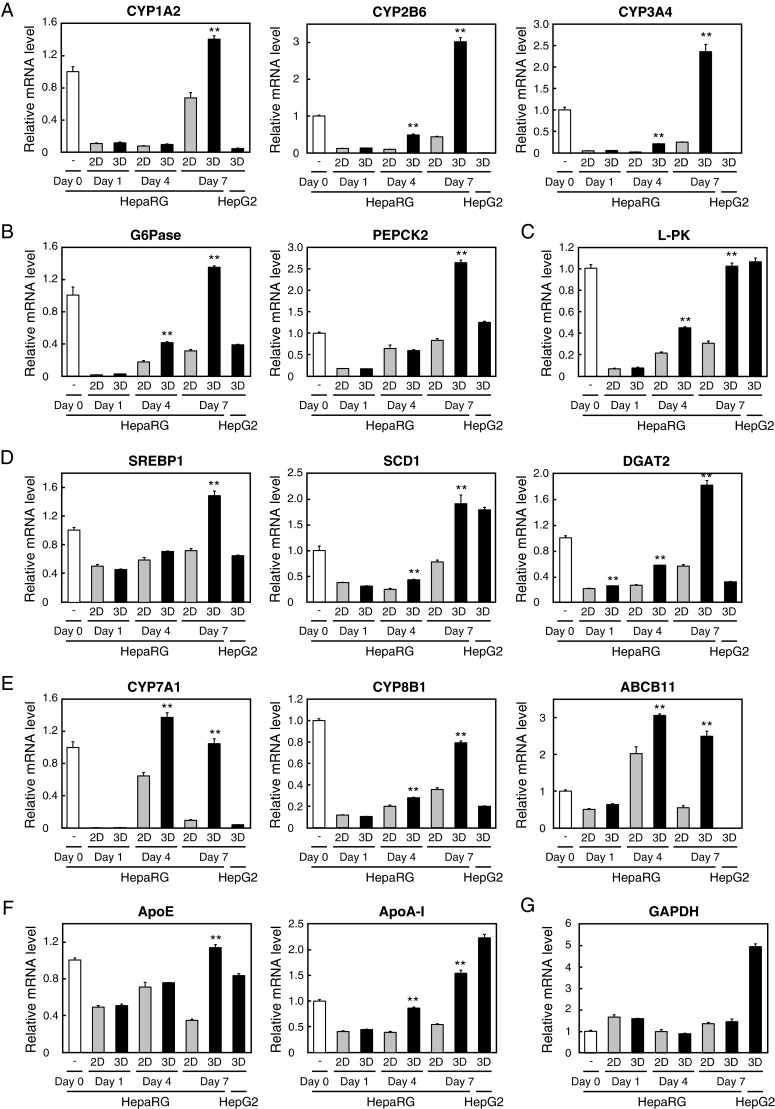
Liver-specific gene expression was augmented in 3D HepaRG spheroids HepG2 and HepaRG cells were seeded into 96-well plates at 2000 cells/well (HepG2), 72000 cells/well (2D, HepaRG) or 24000 cells/well (3D, HepaRG) and subsequently cultured for up to 7 days. Cells were harvested at indicated days: Relative mRNA levels of genes involved in drug metabolism (**A**), gluconeogenesis (**B**), glycolysis (**C**), energetic lipid synthesis (**D**), bile acid metabolism (**E**) and lipoprotein metabolism (**F**) and levels of GAPDH (**G**) were determined by quantitative RT-PCR and were normalized to levels of 18s rRNA. All assays were performed in triplicate. Error bars indicate ± S.E.M. **P<0.01, compared with 2D cultures performed on each day.

Initially, changes in CYP expression of HepaRG cells were assessed by quantitative RT-PCR and CYP1A2, CYP2B6 and CYP3A4 mRNA levels were found to be significantly higher in 3D cultures (Figure 3A). Similar results have been described in previous reports [21,22], although 3D culture systems differ from the hanging drop method. Subsequently, the expression of other genes involved in liver-specific functions was assessed. The mRNA levels for G6Pase (glucose-6-phosphatase) and PEPCK2 (phosphoenolpyruvate carboxykinase 2), both of which are involved in gluconeogenesis, increased at days 4 and/or 7 (Figure 3B). L-PK (L-type pyruvate kinase), one of the rate-limiting glycolytic enzymes, also had higher mRNA levels at days 4 and 7 in 3D cultured spheroids (Figure 3C). Furthermore, the mRNA levels for SREBP1 (sterol regulatory element-binding protein 1), SCD1 (stearoyl-CoA desaturase 1) and DGAT2 (diacylglycerol acyltransferase 2), all of which are involved in energetic lipid synthesis in the liver, also increased at days 4 and 7 (Figure 3D). Similar results were also obtained for the gene expression of CYP7A1, CYP8B1 and ABCB11 (ATP-binding cassette, sub-family B (MDR (multidrug resistance)/TAP (transporter associated with antigen processing)), member 11), all of which are involved in bile acid metabolism (Figure 3E). The expression of ABCB11 was almost undetectable in HepG2 spheroids, as well as CYP2B6 and CYP3A4. The mRNA levels for apoE and apoA-I, which are components of LDL and HDL (high-density lipoprotein) respectively, were also higher at days 4 or/and 7 (Figure 3F). On the other hand, the expression of GAPDH (glyceraldehyde-3-phosphate dehydrogenase), a housekeeping gene, was not statistically different between 2D and 3D cultures (Figure 3G). In this experiment, whereas HepaRG cells were seeded at 24000 cells/40 μl/well in the 3D plates, similar results were also obtained at a cell count of 8000 cells/40 μl/well (result not shown). These results indicate that the 3D hanging drop culture system improved the extensive expression profiles of genes essential for hepatic functions.

### Increase in CYP expression in 3D HepaRG spheroids with treatment of CYP inducers

Last, the change in gene expression of CYP enzymes when HepaRG cells or spheroids were treated with corresponding inducers was assessed. Omeprazole, phenobarbital and rifampicin were selected as inducers for CYP1A2, CYP2B6 and CYP3A4 respectively. When redifferentiated HepaRG cells were treated with CYP inducers, the expression of CYP1A2, CYP2B6 and CYP3A4 increased both in 2D and 3D conditions (Figure 4). These gene expressions were significantly higher in 3D cultured spheroids and also in the inducer-treated group, demonstrating that it is possible to perform CYP induction assays in conditions more closely mimicking physiological environment using the 3D culture plates.

**Figure 4:**
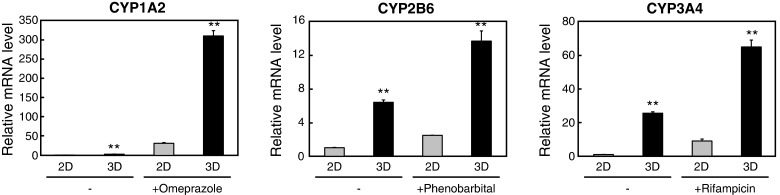
Gene expression of CYP family enzymes increased in 3D HepaRG spheroids treated with CYP inducers HepaRG cells were seeded into 96-well plates at 72000 cells/well (2D) or 24000 cells/well (3D). After 5 days of culture, cells were treated with 100 μM omeprazole, 1000 μM phenobarbital or 20 μM rifampicin for 48 h. Cells were subsequently harvested and relative mRNA levels of CYP1A2, CYP2B6 and CYP3A4 were determined by quantitative RT-PCR and normalized to levels of 18s rRNA. All assays were performed in triplicate. Error bars indicate ± S.E.M. **P<0.01, compared with 2D cultures.

## DISCUSSION

In the present study, we assessed the effect of 3D cultures on hepatocellular characteristics and validated the hypothesis that 3D cultures can improve levels of apoB secretion per viable cell and mRNA levels of various genes exclusively expressed in the liver. We chose Insphero's 96-well plate, which can generate tissue-mimicking spheroids at high throughput. It is noteworthy that this 3D culture system requires neither special biomaterials such as basement membrane proteins nor particular equipment such as bioreactors, thereby the effect of cultural environment change itself can be directly evaluated without any external factors. For instance, Matrigel, an artificial extracellular matrix gel used for 3D cultures, has been reported to contain multiple growth factors, including basic fibroblast growth factor, epidermal growth factor, insulin-like growth factor, transforming growth factor β, platelet-derived growth factor and nerve growth factor [23].

In the quantitative RT-PCR experiment, we assessed many genes responsible for liver function in HepG2 and HepaRG cells. Expression of some critical genes such as CYP3A4 and ABCB11 was very low or almost undetectable in HepG2 cells even when cultured in 3D conditions (Figure 3), indicating that they have limited potential as functional liver cells. In other words, it seems 3D culture cannot substantially aid in the up-regulation of gene expression of cells whose capacities have been somewhat biased. Hence, the use of HepG2 cells should be examined carefully according to each experimental purpose.

On the other hand, we showed that mRNA levels for many critical genes in the liver were generally increased in 3D cultures of HepaRG cells (Figure 3). The genes picked up in the analysis are known to be directly regulated by transcription factors highly expressed in the liver. For instance, CYP3A4, PEPCK2, L-PK, SCD1, ABCB11 and ApoA-I are target genes for PXR (pregnane X receptor) [24], FOXO1 (forkhead box O 1) [25], ChREBP (carbohydrate responsive element-binding protein) [26], SREBP1 [27], FXR (farnesoid X receptor) [28] and LRH-1 (liver receptor homologue-1) [29] respectively. The data suggest that these transcription factors are activated by changing the cultured environment from 2D to 3D.

Moreover, mRNA levels of genes with contradictory functions such as G6Pase/L-PK and apoE/apoA-I were augmented in 3D cultures (Figures 3B, 3C, 3F). This suggests that basal metabolic rate of HepaRG cells would be enhanced in 3D conditions. By contrast, the expression of genes involved in cholesterol metabolism (e.g. LDL receptor) or fatty acid oxidation (e.g. CTP1A, carnitine palmitoyltransferase 1A) did not increase in the 3D cultured spheroids (result not shown). The results indicate that the change in cellular capacity triggered by 3D cultures is seemingly somewhat biased. Further investigation would be needed to classify activated pathways responsible for hepatocellular functions.

We also showed that the expression of numerous genes was significantly higher at day 7 in 3D cultured HepaRG spheroids compared with day 0, a manufacturer-supplied fully differentiated state from 2D cultures, implying that 3D cultures enhance the hepatic activity of HepaRG cells. This suggests that it would be possible by employing this 3D culture system to conduct assays which would otherwise be difficult to implement for reasons such as having cellular responses too low to detect.

Although we performed 3D cultures for single type of cells in the present study, it would be of great interest to try co-culturing with different types of cells. It is reported that HUVEC (human umbilical vein endothelial cell) predominantly migrate to the periphery, representing a natural cell-type composition, when it is cultured together with HepG2 cells using the hanging drop method [14]. Since the liver is composed of numerous types of cells, such as parenchymal hepatocytes, Kupffer cells, macrophages and hepatic satellite cells, combining these cells in a single hanging droplet may be effective to generate more physiological microtissues.

In summary, to the best of our knowledge, this is the first report of evidence that 3D spheroid cultures up-regulate apoB production and the extensive liver-specific gene expression of cultured cells. From these evidences, one can expect that diverse metabolic functions are improved by 3D cultures and therefore they should be examined by establishing functional assays including metabolite measurement, which may provide a useful system for drug discovery research in the future.

## Abbreviations

ABCB11: ATP-binding cassette, sub-family B (MDR/TAP), member 11
Apo: apolipoprotein
2D: two-dimensional
3D: three-dimensional
CYP: cytochrome P450
DMEM: Dulbecco's modified Eagle's medium
GAPDH: glyceraldehyde-3-phosphate dehydrogenase
G6Pase: glucose-6-phosphatase
PHH: primary human hepatocyte
LDL: low-density lipoprotein
L-PK: L-type pyruvate kinase
PEPCK: phosphoenolpyruvate carboxykinase
RT: reverse transcription
SREBP1: sterol regulatory element-binding protein 1
SCD1: stearoyl-CoA desaturase 1
VLDL: very low-density lipoprotein
